# Letting Work What Works—Effectively Preventing Juvenile Delinquency in the Netherlands: A Meta-Analysis of the Evidence

**DOI:** 10.1177/0306624X251344620

**Published:** 2025-08-16

**Authors:** Puck Meulen, Naomi Koning, Mark Assink, Susan van Hooren, Emile Kolthoff, Evelyn Heynen

**Affiliations:** 1 Open University of The Netherlands, Heerlen, The Netherlands; 2 University of Amsterdam, The Netherlands; 3 Dutch Innovation Network for Societal Youth Challenges, Garage2020, Amsterdam, The Netherlands; 4 Zuyd University of Applied Sciences, Heerlen, The Netherlands

**Keywords:** youth interventions, evidence-based, prevention, juvenile delinquency, meta-analysis

## Abstract

This meta-analysis evaluated judicial interventions for juvenile delinquency certified by the Netherlands Youth Institute with “initial indications” for effectiveness. Ten (quasi-)experimental studies examining TOOLS4U, Multidimensional Family Therapy (MDFT), Multi Systemic Therapy (MST), Only You Decide who you Are (OYD), Responsive Social Skills Therapy (Re-SST), and Responsive Aggression Regulation Therapy (Re-ART) were synthesized. The overall effect was small, positive, and significant (*g* = 0.22; 95%CI: 0.02, 0.42). Responsive cognitive behavioral therapy (Re-SST and Re-ART) and the sports-based intervention OYD seem effective, whereas social skills training (TOOLS4U) and family-based systemic interventions (MDFT and MST) seem ineffective. However, effectiveness of family-based programs can possibly be enhanced if youth at medium-to-high risk for delinquency is treated for 6 months or longer, which aligns with the Risk-Need-Responsivity model. Better program delivery and general instead of specific offenses were associated with larger effects. We advocate a stronger evidence-based RNR approach to juvenile delinquency in the Netherlands.

## Plain language summary

Effectively preventing juvenile delinquency in the Netherlands

Juvenile delinquency remains a serious issue in the Netherlands. Preventive interventions have proliferated, but many lack strong theoretical foundations and research support. The Netherlands Youth Institute (NYI) continuously monitors all available evidence for intervention effectiveness and certifies those showing initial indications of effectiveness. This study analyzed that evidence to better understand the overall effectiveness of preventive interventions against Dutch juvenile delinquency. Only six interventions currently offered to Dutch juveniles were certified by the NYI and reported on delinquency: TOOLS4U, Multidimensional Family Therapy (MDFT), Multi Systemic Therapy (MST), Only You Decide who you Are (OYD), Responsive Social Skills Therapy (Re-SST), and Responsive Aggression Regulation Therapy (Re-ART). Their combined effectiveness proved to be small. Responsive cognitive behavioral therapy (Re-SST, Re-ART) and the sports-based intervention OYD seem effective, while social skills training (TOOLS4U) and family-based systemic interventions (MDFT, MST) seem ineffective. However, family-based program effectiveness may improve if youth at medium-to-high delinquency risk are treated for at least six months. Better program delivery and targeting general rather than specific offenses yielded larger effects. Practitioners should emphasize an evidence-based approach to effectively prevent Dutch juvenile delinquency and make research-informed decisions. The Dutch national quality framework, NYI-certified interventions, and Risk-Need-Responsivity model principles are the key to strengthening practice. Since many of the included interventions are also implemented internationally, this research is of international importance.

## Introduction

Juvenile delinquency in the Netherlands, which is defined as criminal behavior committed by young people up to the age of 24, has decreased about 50% between 2010 and 2023, and particularly among 12- to 23-year-old suspected and convicted juvenile offenders (Kessels, 2023; Tollenaar & Van der Laan, 2023). This decline is reflected in a decrease of self-reported delinquency from 41.2% to 34.7% between 2005 and 2015 (Van der Laan et al., 2019, 2024). However, the number of Dutch minor offenders involved in serious violent crimes significantly increased between 2016 and 2021: 782 versus 535 minors were involved in property crimes, 193 versus 139 in serious assaults, and 72 versus 31 in (attempted) manslaughter (Mcgloin & Piquero, 2009; Tollenaar & Van der Laan, 2023; Van Mastrigt & Carrington, 2019). These numbers continued to increase—although slightly—in 2022 and 2023, according to preliminary data (Van der Laan et al., 2023).

Although most youths will outgrow delinquent behavior without treatment, some—particularly high-risk youths—do need behavioral interventions to prevent persistent juvenile delinquency (Moffitt, 1993), which is underlined by the theoretical principles of the *Risk, Need, and Responsivity (RNR) model* (Andrews et al., 2011; Bonta & Andrews, 2023; Boone, 2013; Van der Laan et al., 2010; Van der Put et al., 2011). Such interventions refer to theoretically grounded, systematic, and goal-oriented behavioral approaches for youth at substantial risk of delinquency (and differ from universal prevention that targets all young people; Netherlands Youth Institute (NYI), 2024). The intensity of these interventions is defined in terms of duration, frequency, and invasiveness of professional care. The interventions should target dynamic (modifiable) risk and protective factors for juvenile delinquency at the level of the youth, their caregivers, and/or the caregiving environment.

### Literature Review

**Figure A1.**
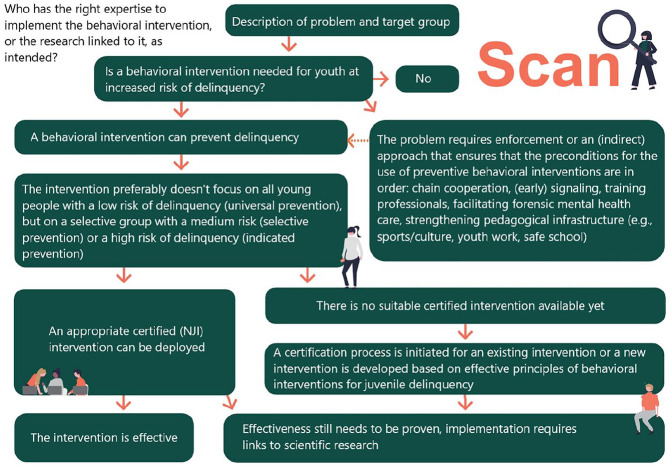
Infographic about which steps municipalities should take in deciding about behavioral interventions to prevent juvenile delinquency, based on the national quality framework (Hendriks & Stams, 2024).

The likelihood of engagement in delinquent behavior increases with the severity, and especially the number of criminogenic risk factors (Assink et al., 2015; Bonta & Andrews, 2023). In case of only a few risk factors, selective prevention (targeting groups at higher risk) may be sufficient, but indicated preventive interventions (for individuals with a substantial risk of serious and persistent delinquency) are required when many risk factors co-occur (Assink et al., 2015). If preventive behavioral intervention is required, two situations may arise: (a) A certified theory-based behavioral intervention that has either been proven effective in (quasi-)experimental research or is awaiting proof of effectiveness can be used, or (b) No suitable certified intervention is available, requiring either the certification of an existing one that seems promising from a theoretical perspective, or a new intervention should be developed based on the effective principles of judicial interventions (Bonta & Andrews, 2023), while its effectiveness still needs to be proven (see Figure A1 for required steps in both situations).

In the Netherlands there has been a proliferation of indicated preventive interventions for juvenile delinquency. Many of these are uncertified, and lack robust theoretical foundations and strong empirical support from studies with an experimental design (Mulder et al., 2023; Spanjaard, 2019). This hampers professionals in making the appropriate choice for a preventive behavioral intervention that matches the risk and needs of a juvenile (Hendriks & Stams, 2012). This creates a need to invest only in evidence-based interventions that have proven to be effective in empirical research, and in such empirical research itself. In the Netherlands, these interventions should be continuously monitored and should receive certification of the “Judicial Interventions” committee of the Netherlands Youth Institute which is assigned to evaluate and certify interventions for juvenile delinquency (Hendriks & Stams, 2024; Netherlands Youth Institute (NYI), 2024).

Recently, a Dutch national quality framework highlighted the need for evidence-based guidelines for (local) governments to effectively prevent juvenile delinquency in the Netherlands (Hendriks & Stams, 2024). Drawing on these guidelines, preventive behavioral interventions should at a minimum be certified as effective based on preliminary evidence, be (cost)effective, beneficial for juveniles and society, serve therapeutic aims (positive, supportive, constructive, and meeting basic needs of self-determination), and align with children’s rights (Cohen, 1998; Cohen & Piquero, 2009; De Jong, 2022; De Vries et al., 2015; Farrington et al., 2017; Greenwood, 2008; Lipsey, 2009; Reil et al., 2021; Van der Helm et al., 2018; Weerman et al., 2022; Weijers & Eliaerts, 2015; Weisburd et al., 2016). Notably, article 12 of the Convention on the Rights of the Child (CRC) emphasizes participation and the right of a child to be heard, which is grounded in self-determination. Article 16 of the CRC underscores privacy rights, and the need for empirical evidence to ensure interventions achieve their intended outcomes without harming the child. Only under these conditions, the use of preventive interventions seems to be legitimated (Hendriks & Stams, 2024), and socially, ethically, and legally justifiable. By contrast, coercive and disciplinary interventions are unsuitable for preventing juvenile delinquency (Weisburd et al., 2001). The current meta-analysis builds on the quality guidelines of Hendriks and Stams (2024) by examining to what extent evidence-based behavioral interventions for juvenile delinquency that have been certified by the NYI are truly effective in the Netherlands.

The literature suggests that preventive behavioral interventions are effective if they are in line with the *Risk, Need, and Responsivity (RNR) model* (Andrews et al., 2011; Bonta & Andrews, 2023; Boone, 2013; Van der Laan et al., 2010; Van der Put et al., 2011). This model comprises three principles and states that an intervention’s intensity should be tailored to the risk of delinquency, and that risk assessment should be based on validated and reliable instruments (*Risk principle*). It also states that interventions should target modifiable criminogenic needs (*Need principle*), and that interventions should include tailored (potential) effective techniques and elements (such as cognitive behavioral therapy (CBT) elements), and take the (treatment) motivation and other individual characteristics of the youth and his/her social environment into account (*Responsivity principle*).

The RNR model has been criticized for emphasizing risk rather than protective factors (Ward & Maruna, 2007). However, RNR-based interventions that target criminogenic needs mostly address both criminogenic risk and protective factors (Lösel & Farrington, 2012), whereas focusing solely on well-being and strengths—like in the Good Lives Model (GLM; Ward et al., 2007)—may fail to sufficiently address the root causes of delinquency (Andrews et al., 2011). Moreover, risk factors tend to be more pervasive than protective factors (Baumeister et al., 2001; Vanderbilt-Adriance & Shaw, 2008) and should therefore be the primary target of (preventive) interventions for juvenile delinquency. Despite criticisms, the RNR framework seems to be an appropriate and the most comprehensive theoretical model to explain how delinquency develops from a life course and systemic perspective, and which criminogenic factors should be targeted in (judicial) interventions to prevent delinquency, using well-established principles of cognitive-behavioral therapy in treatment that is tailored to individual differences (Assink et al., 2015; De Vries et al., 2015; Moffitt, 1993; Sampson & Laub, 1993; Spruit et al., 2017).

In addition to the RNR principles, preventive behavioral interventions tend to be more effective when implemented as intended (*program integrity principle*), delivered by adequately skilled and trained professionals (*professionalism principle*), and in the youth’s natural living environment instead of a residential institution (*in-society principle*; Goense et al., 2016; Van der Laan et al., 2010). Next to this, interventions may appear more effective than they actually are, when weaker research designs with low internal validity are used (Weisburd et al., 2001, 2016). Interventions can also have effects that fail to have clinical significance or may even be harmful (e.g., Dishion et al., 1999; Petrosino et al., 2003), in particular if coercion is high (Parhar et al., 2008).

In the Netherlands, the NYI is a governmental organization that monitors the available evidence for the effectiveness of judicial behavioral interventions for juvenile delinquency offered in the Netherlands. The NYI “Judicial Interventions” certification committee certifies these interventions according to four “levels” of evidence available for intervention effectiveness: (1) Interventions can be *theoretically founded* (currently 16; see Table A1), implying that intervention developers have clearly described the problem(s), risk(s) or theme(s) their intervention addresses, the target group, objectives, approach, and conditions of the intervention. Interventions classified at this level usually still need to be empirically investigated. A process evaluation is required to acquire this certification level. Interventions are classified into the next level, (2) *initial indications of effectiveness* (currently 9; see Table A2), when two (or more) pre-post studies are available. At least one of these studies must have been conducted in the Netherlands. When Dutch research that provides relatively strong evidence is available, such as studies with a control group, one study is sufficient to acquire this certification level. Interventions are classified into the next level, (3) *good indications of effectiveness* (currently 1; see Table A3), when two studies providing relatively strong evidence are available, of which at least one must have been conducted in the Netherlands. Also sufficient is a single Dutch study with strong to very strong evidence, such as a (randomized) controlled trial with a follow-up measurement, or a repeated case study involving at least six cases across different intervention settings or at least ten cases monitored in one intervention setting. Interventions are classified into the highest level, (4) *strong indications of effectiveness* (currently 0), when two (or more) studies are available that provide strong to very strong evidence for intervention effectiveness, such as a (randomized) controlled trial with a control group and a follow-up measurement. At least one study must have been conducted in the Netherlands. Alternatively, a repeated case study with at least ten cases monitored in different intervention settings is also sufficient. Besides *judicial* interventions, the NYI has classified 36 *non-judicial preventive or curative* interventions for externalizing problems into one of the four levels (see Table A4).

### Current Study

The current study is particularly relevant given our limited (meta-analytical) understanding of the effectiveness of preventive judicial behavioral interventions for juvenile delinquency in the Netherlands, due to inconsistencies in evaluation methods, shortage of (quasi-)experimental studies, and the frequent omission of delinquency/recidivism as an outcome measure in primary research. Only ten interventions that are currently being offered to Dutch juveniles (and their family members) were certified by the NYI certification committee with at least *initial indications of effectiveness* in preventing juvenile delinquency. This meta-analysis adds knowledge by examining the overall effectiveness of these ten interventions by synthesizing results from primary (quasi-)experimental studies on delinquency/recidivism. A further aim was to test sample, study design, publication, program, and outcome characteristics as moderators of the overall effectiveness to get a grasp of the circumstances in which effectiveness increases or decreases. Since many of these interventions are also implemented in other countries than the Netherlands, this meta-analysis is of international importance.

## Methods

In conducting and reporting the current meta-analysis, the 2020 Preferred Reporting Items for Systematic reviews and Meta-Analyses (PRISMA) guidelines were followed (Page et al., 2021a, 2021b). In addition, the reporting guideline for synthesis without meta-analysis (SWiM) in systematic reviews was used (Campbell et al., 2020). The protocol for study screening and selection was registered in PROSPERO (registration number: 562525).

### Inclusion Criteria

Four criteria for study inclusion were formulated. First, studies had to evaluate a judicial behavioral youth intervention as defined by the NYI (see Introduction). Second, the intervention is specifically aimed at preventing juvenile delinquency and criminal recidivism, and not behavioral problems. Therefore, studies had to report on juvenile delinquency and/or recidivism. We excluded meta-analyses as well as studies that focused solely on changes in risk factors, protective factors, behavioral problems or recidivism risk. Third, the intervention had to be certified by the NYI with at least *initial indications of effectiveness* (i.e., level 2 of their classification system), as this level of classification indicates that some research on the intervention’s effectiveness has been conducted. Fourth, studies had to be performed in the Netherlands as we were specifically interested in the impact of interventions on Dutch populations of juvenile offenders and at-risk juveniles.

### Study Selection

The “Database Effective Youth Interventions” (Database Effectieve Jeugdinterventies) of the NYI contains evidence-based and certified behavioral youth interventions, each with a detailed description of the intervention target group, goal, approach, materials used, theoretical basis, and scientific (effectiveness) research. Hendriks and Stams (2024) provide an overview of these interventions specifying whether interventions are (non-)judicial, whether they are curative or aimed at preventing (a specific type of) juvenile delinquency/recidivism or behavioral problems, and if and at what level the interventions are certified. The broad interpretation of prevention by Hendriks and Stams (2024; see introduction), might blur the line between curative and preventive interventions. Preventive interventions aim to prevent juvenile delinquency, while curative interventions focus on reducing delinquency in youth with existing problem behavior (Netherlands Youth Institute (NYI), 2024).

The overview of Hendriks and Stams (2024), based on the NYI “Database Effective Youth Interventions,” was used to select all judicial behavioral youth interventions (first inclusion criteria) currently certified by the NYI with at least *initial indications of effectiveness* (third inclusion criteria). The database was first screened for any developments in certification levels of interventions after the publication of Hendriks and Stams (2024). This resulted in ten interventions.

All Dutch primary studies that were referred to in the NYI database until March 2025 and examined the effectiveness of the ten interventions were first identified. Next, a total of forty-three titles and abstracts were screened after which twenty-one studies were excluded because they assessed risk factors, protective factors, or behavioral problems other than delinquency/recidivism. An additional nine studies were excluded as recidivism risk was assessed, and three meta-analyses were excluded as well. In the end, ten studies were included. Four studies were coded together with another study, since they reported on the same intervention and the same sample.

**Figure 1.**
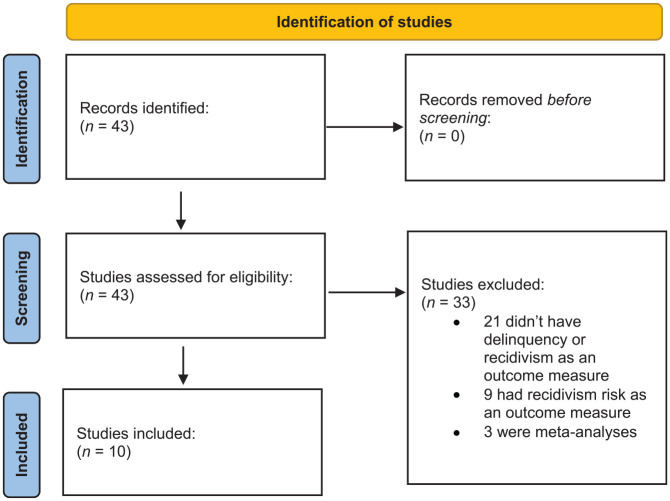
Flowchart of study selection procedure, following the preferred reporting items for systematic review and meta-analysis (PRISMA).

This resulted in six studies that were eligible for inclusion, from which sixty-five effect sizes could be extracted (see Figure 1). The included studies (marked with an asterisk in the reference list) examined six of the ten judicial behavioral youth interventions with at least *initial indications of effectiveness*: TOOLS4U, Multidimensional Family Therapy (MDFT), Multi Systemic Therapy (MST), Only You Decide who you Are (OYD), Responsive Social Skills Therapy (Re-SST), and Responsive Aggression Regulation Therapy (Re-ART) for Young Adults. The interventions Forensic Outpatient System Therapy, Parenting with Love and Boundaries, Responsive Aggression Regulation Therapy (Re-ART) for Youth, and Aggression Replacement Training (ART) could not be included, as effectiveness studies assessing delinquency/recidivism in a Dutch sample of delinquent youths were unavailable for these interventions.

Forensic Outpatient System Therapy is a 3-9 months lasting outreach treatment that combines a systemic and CBT approach with the Nonviolent Resistance method, addressing youth-specific, family, and systemic factors. Parenting with Love and Boundaries uses a systemic, solution-focused, and CBT approach to restore family relationships and improve parenting skills in individual family sessions and six group meetings, with the aim to reduce behavioral problems and recidivism risk. Re-ART for Youth is a 4 to 18 months lasting primarily individual treatment using a CBT approach and structured do-exercises (drama therapy, experiential techniques, mindfulness), with a continuous focus on strengthening the treatment motivation (Netherlands Youth Institute (NYI), 2024).

ART is a 3.5 to 5 months lasting multimodal CBT intervention (addressing social skills, anger control, and moral reasoning) that teaches youth to respond prosocial and with self-control when they experience anger (Netherlands Youth Institute (NYI), 2024). Notably, ART is the only intervention certified with *good indications of effectiveness* (i.e., level 3) in preventing juvenile delinquency, for which—following the NYI criteria—at least one Dutch study that can provide relatively strong evidence must be available (e.g., a study with a control group). However, none of the Dutch ART effectiveness studies report delinquency/recidivism outcomes, one study lacks a control group (Smeets et al., 2016), another uses a non-comparable control group (Hornsveld & Kraaimaat, 2011), and one is a meta-analysis on CBT rather than ART (Smeets et al., 2015). Thus, the certification of ART with *good indications of effectiveness* appears unjustified, and the available ART effectiveness studies do not meet the inclusion criteria of the current meta-analysis.

At this point, it is worth stating that the NYI certification of Forensic Outpatient System Therapy, Parenting with Love and Boundaries, and Re-ART for Youth with *initial indications of effectiveness* can be questioned. The certification of these interventions is based on primary research that did not measure delinquency/recidivism directly, but reported on delinquency-related outcomes, such as risk and protective factors, (general) behavioral problems, or recidivism risk. Also, it seems that interventions are certified based on single positive—and sometimes small—effects, even when overall effects across outcomes or follow-up assessments are non-significant. In contrast, the current meta-analysis applied a more stringent effectiveness test, and specifically synthesized delinquency/recidivism outcomes that were assessed directly after the intervention and/or in follow-up assessments.

### Study Coding

A coding scheme was developed for coding all studies. Multiple sample, study design, publication, program, and outcome characteristics were coded in IBM SPSS Statistics (Version 29). Variables not reported in studies, were left blank in the dataset. Regarding *publication characteristics*, title, publication year and type, author names, journal name, and journal impact factor were coded. As for *study design characteristics*, study design (randomized controlled trial, quasi-experimental, no control group), type of control group (no treatment, treatment as usual, placebo, multiple baseline), matched (yes/no), intention to treat (yes/no), and follow-up (post-test, follow-up assessment) were coded. Study quality was assessed with an index (Conley et al., 2022) that is derived from the Cochrane Collaboration’s risk of bias tool (Downs & Black, 1998; Higgins et al., 2011; Thomas et al., 2004). This index assesses 10 features of studies: whether or not a study is peer reviewed and journal impact factor, type of study design, author involvement in intervention development, reporting on participant characteristics, sample size, study completion rate, reliability of measures, validity of measures, adjustment for pretest differences, and whether or not intent-to-treat analyses were performed. Each feature is scored from 0 (lowest quality) to 3 (highest quality), with a summed score of 20 indicating average research practices according to Conley et al. (2022).

As for *sample characteristics*, sample size, intervention group and control group size, percentage of attrition, percentage of non-response, mean age and age range of the sample, percentage of boys, and proportion ethnic minority were coded. Regarding *program characteristics*, program target (externalizing, internalizing, psychosocial difficulties, substance use, distorted cognitions, cognitive beliefs, deviant peer association, peer problems, parenting/family functioning, skills, social support, structured free time), intervention name, treatment design (group therapy, individual therapy, both), program integrity (high, low, medium, not reported), treatment duration (in months), and total number of treatment sessions were coded. Concerning *outcome characteristics*, offense type (general, violence, property, drugs, minor, vandalism, sexual, status, other), how delinquency was operationalized (arrest, convicted, court or police contacts, arrest and convictions), type of delinquency assessment (official record, questionnaire self-report, questionnaire other-report, clinical interview), and outcome type (percentage, number, time to re-offense, severity of offense) were coded.

### Calculation of Effect Sizes

Hedges’ *g* was chosen as the common effect size estimate. Hedges’ *g* is the standardized difference between the means of an intervention group and a control group, and controls for small sample sizes (Hedges, 1981; Hedges & Olkin, 1985). In extracting and calculating effect sizes, it was important that effects were properly expressed in Hedges’ *g* values, which could be either positive or negative. A positive Hedges’ *g* indicated that the intervention group outperformed the control group (Hedges & Olkin, 1985), whereas a negative sign was assigned when the control group performed better.

Formulas of Borenstein et al. (2009), Cohen (1988), Hedges and Olkin (1985), Lipsey and Wilson (2001), Ruscio (2008), and Wilson (2023) were used to calculate *g* whenever these values could not be extracted directly from an included study. To apply these formulas, the “Effectsize: Indices of effect size” R package was used (Ben-Shachar et al., 2020). When studies only reported that an effect size was non-significant, we conservatively included a null effect, following Mullen (1989). This procedure was preferred above excluding primary studies, as the latter would reduce the statistical power in the analyses (Conley et al., 2022).

### Statistical Analyses

The meta-analysis was conducted in R version 4.3.2 (R Development Core Team, 2023), in which a three-level approach to meta-analysis was applied. This approach can be used to model dependency of effect sizes, given that multiple relevant effect sizes could be extracted from most included studies (Assink & Wibbelink, 2016; Cheung, 2014; Konstantopoulos, 2011). A three-level meta-analysis considers three variance components distributed over the three levels of the model to model effect size dependency: sampling variance of the extracted effect sizes (level 1); variance between effect sizes extracted from the same study (level 2); and variance between studies (level 3; Assink & Wibbelink, 2016; Cheung, 2014; Konstantopoulos, 2011; López-López et al., 2014). This approach allows for extraction of all relevant effect sizes from individual studies, so that all information is preserved, and maximum statistical power in the statistical analysis is achieved (Assink & Wibbelink, 2016). It also allows for more moderator analyses than traditional (two-level) meta-analysis, as more information is available in the synthesis (Cheung, 2014).

To determine whether the variance at the second and/or third level of the model was significant, a one-sided log-likelihood-ratio test was performed for each level of the model (Assink & Wibbelink, 2016). This test compares the deviance of the full model to the deviance of a model excluding the (within-study or between-study) variance component. Significant variance distributed at the second and/or third level indicates heterogeneity in the effect size distribution, meaning that the overall mean effect size is not a correct estimate of the true/population effect. In case of heterogeneity, we proceeded with moderator analyses in attempts to identify which of the coded sample, study design, publication, program, and/or outcome characteristics may explain this heterogeneity.

We used the rma.mv function from the metafor package (Viechtbauer, 2010) in R version 4.3.2 (R Development Core Team, 2023), following the setup and syntax described by Assink and Wibbelink (2016), to model the effect size dependency (Van den Noortgate et al., 2013, 2015). The overall effect was estimated with an intercept-only model, while the coded variables were tested as moderators in bivariate models. The *t* distribution was used for testing individual regression coefficients, and the calculation of 95% confidence intervals (Knapp & Hartung, 2003). Model parameters were estimated with the restricted maximum likelihood estimation method. Before conducting moderator analyses, continuous variables were centered around their means, and dichotomous dummy variables were created for categories of discrete variables (Tabachnik & Fidell, 2013). The significance level was set to .05 in all analyses.

For clinical importance, we converted effect sizes (*g*) into percentages of change of the intervention group, which is designated as the Success Rate Difference (SRD). This conversion was done with the formula of Kraemer and Kupfer (2006) that transforms Hedges’ *g* (Standardized Mean Difference; SMD) into the Area Under Curve (AUC) statistic, and subsequently provides a percentage of change or SRD ([2 * AUC – 1) * 100]).

### Bias Assessment

Risk of bias was addressed by testing the index of study quality as a moderator, and by comprehensive publication and selection bias analyses. Studies producing non-significant or negative results are less likely to be published than studies producing significant and positive results (Beelmann & Lösel, 2021; Lipsey & Wilson, 2001; Olsson et al., 2021; Rothstein, 2008), which is referred to as publication bias or the file drawer problem (Rosenthal, 1995). To determine to what extent the results were affected by bias we first performed the adjusted three level Egger’s regression test (Egger et al., 1997) as described by Fernández-Castilla et al. (2020, 2021). This concerns a test of the association between effect sizes and their standard errors, taking into account the effect size dependency. A significant association is indicative of bias. We also performed the trim-and-fill analysis to examine funnel plot symmetry (Fernández-Castilla et al., 2020, 2021), which is based on the assumption that the effect size distribution is symmetrical when bias is absent. In this technique, the estimated number of effect sizes in the left or right side of the effect size distribution is related to a cut-off value of the trim-and-fill method estimator. If this number exceeds the cut-off value, this may indicate publication or selection bias. Finally, the moderating effects of journal impact factor and publication year of studies were tested. A significant effect indicates that publication bias and/or a declined effect (over time) may exist, respectively.

## Results

### Description of the Included Interventions

A total of ten studies examining six interventions were included in this meta-analysis (see Table A5 for an overview). Five interventions are curative (for juvenile offenders). *TOOLS4U* is a program offered to individual juveniles that teaches them cognitive and social skills in 12 to 15 75-min sessions, with an optional 4-session parent component focused on positive communication, supervision, and problem-solving (Van der Stouwe et al., 2018, 2019; Van der Stouwe, 2020). *Multidimensional Family Therapy* involves sessions with juveniles and their parents to help juveniles avoid problems and achieve personal goals, while addressing caretakers and family members to improve parenting and family bonds (Van der Pol et al., 2018, 2021). *Multi Systemic Therapy* is a four- to five-month home- and family-based treatment, which aims to reduce behavioral problems, enhance family problem-solving, and improve caregivers’ parenting skills. Juveniles engaged in this therapy have 24/7 access to a therapist that can intervene in key social systems surrounding the juvenile. In addition to preventing general delinquency and recidivism, it demonstrates effectiveness in reducing property crime (Asscher et al., 2013, 2014).

*Responsive Social Skills Therapy* improves social and cognitive skills, problem-solving, decision-making, and confidence. It is offered individually, in groups of juveniles, or in a combination of individual and group sessions for at least 1 to 1.5 hours weekly, lasting four to fourteen months (Hoogsteder et al., 2021). *Responsive Aggression Regulation Therapy for Young Adults* comprises primarily CBT offered to individual juveniles with practical exercises and a strong motivational counseling style that lasts between 5 and 18 months, depending on the learning pace and severity of problems. A substantial part of the intervention consists of experiential techniques. Next to preventing general delinquency and recidivism, it also demonstrates effectiveness in reducing property and violent crime (Hoogsteder et al., 2018). Next to these five curative interventions, one intervention is preventive. *Only You Decide who you Are* is a one year structured leisure activity program in which juveniles take part in a team sport to strengthen protective factors and reduce risk factors for delinquent behavior (Gubbels et al., 2018).

### Overall Effect and Effect Size Heterogeneity

As shown in Table 1, the overall effect of the interventions with *initial indications of effectiveness* according to the NYI on juvenile delinquency was *g* = 0.22 (*p* = .032; 95% CI: 0.02, 0.42), which is significant and small in magnitude (Durlak, 2009; Gaeta & Brydges, 2020). This effect translates to a reduction of 12.36% in juvenile delinquency.

**Table 1.** Overall Effect of Interventions With Initial Indications of Effectiveness on Juvenile Delinquency and Recidivism.

*k*	#ES	M *g*	95% CI	*p*	σ^2^_level 2_	σ^2^_level 3_	% var. level 1	% var. level 2	% var. level 3
6	65	0.22	0.02, 0.42	.032	.016**	.055***	26.70	16.28	57.02

*Note. k* = number of studies; #ES = number of effect sizes; M *g* = mean effect size (Hedges’ *g*); CI = confidence interval; σ^2^_level 2_ = variance between effect sizes of the same study; σ^2^_level 3_ = variance between studies; % Var = percentage of variance distributed.

** *p* < .01. ***p < .001.

**Figure A2.**
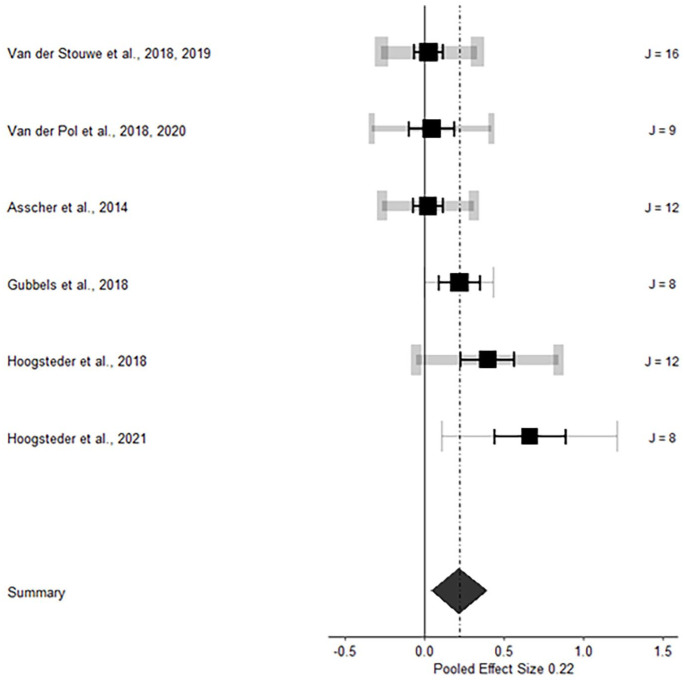
Forest plot of the overall effectiveness of Dutch judicial youth interventions on juvenile delinquency. *Note*. The black line represents a confidence interval of the study’s effect size based on the sample size and total number of effect sizes within each study. The gray line represents an additional confidence interval based on the sampling variance of individual observed effect sizes within a study. The thickness of the gray confidence intervals is proportional to the number of effect sizes reported within studies (Fernández-Castilla et al., 2020). J = number of effect sizes.

The three-level forest plot in Figure A2 indicates how the effect sizes are distributed over the studies. The studies of Gubbels et al. (2018) and Hoogsteder et al. (2018, 2021) only reported positive effect sizes. In contrast, there are different studies (Asscher et al., 2013, 2014; Van der Pol et al., 2018, 2021; Van der Stouwe et al., 2018, 2019; Van der Stouwe, 2020) which reported both positive and negative effect sizes, with smaller positive effects than those reported by Gubbels et al. (2018) and Hoogsteder et al. (2018, 2021). This suggests that Only You Decide who you Are, Responsive Social Skills Therapy, and Responsive Aggression Regulation Therapy for Young Adults are effective in preventing juvenile delinquency and recidivism, while TOOLS4U, Multi Systemic Therapy, and Multidimensional Family Therapy are not effective.

There was significant heterogeneity within studies (σ^2^_level 2_ = .016, *p* < .01), and between studies (σ^2^_level 3_ _=_ .055, *p* < .001). Of the total variance, 16% was distributed at the within-study level, and 57% at the between-study level, while sampling variance was about 27%. In an attempt to explain the significant within- and between study variance in effect sizes, moderator analyses were conducted (see Table 2).

**Table 2.** Results of Bivariate Moderator Analyses.

Moderator variables	*k*	*#ES*	*B*_0_ / *g*	*t*_0_	*B*_1_	*t*_1_	*F* (*df*_1_, *df*_2_)
Outcome characteristics							
Offense type (broad)							*F* (1, 63) = 4.74*
General offenses	6	37	0.28	2.43*			
Specific offenses	5	28	0.14	1.22	−0.14	−2.18*	
Offense type (narrow)							*F* (2, 62) = 2.38
General offenses	6	37	0.28	2.32*			
Property offenses	4	14	0.16	1.22	−0.12	−1.41	
Violent offenses	5	14	0.13	1.08	−0.15	−2.04*	
Delinquency type							*F* (2, 62) = 1.56
Convicted	4	34	0.23	2.19*			
Arrest	4	27	0.19	1.76^+^	−0.04	−0.63	
Self-reported delinquency	1	4	0.38	2.51*	0.15	1.10	
Outcome type							*F* (3, 61) = 0.24
Percentage	4	30	0.25	2.32*			
Number	5	29	0.20	1.87^+^	−0.05	−0.70	
Time to re-offense	3	4	0.25	1.69^+^	−0.00	−0.03	
Severity of offense	1	2	0.13	0.61	−0.12	−0.62	
Sample characteristics							
Age	6	65	0.23	2.10*	0.09	0.97	*F* (1, 63) = 0.94
Gender (percentage of boys)	6	65	0.22	2.25**	0.01	0.45	*F* (1, 63) = 0.21
Program characteristics							
Intervention program							*F* (3, 61) = 7.15***
Responsive CBT (Re-SST, Re-Art)	2	20	0.50	5.94***			
Individual sports intervention (OYD)	4	8	0.22	2.23*	−0.28	−2.18*	
Social skills intervention (TOOLS4U)	1	16	0.03	0.31	−0.47	−3.79***	
Family-based intervention (MDFT, MST)	2	21	0.03	0.47	−0.47	−4.21***	
Program targets^1^							
Externalizing							*F* (1, 63) = 0.72
yes	5	49	0.03	0.12			
no	1	16	0.26	2.28*	0.23	0.85	
Internalizing							*F* (1, 63) = 0.56
yes	5	53	0.40	1.54			
no	1	12	0.19	1.62	-0.21	-0.75	
Psychosocial difficulties							*F* (1, 63) = 2.63
yes	4	37	0.03	0.18			
no	2	28	0.32	2.98**	0.29	1.62	
Substance use							*F* (1, 63) = 2.02
yes	2	21	0.31	2.75**			
no	4	44	0.03	0.22	- 0.28	-1.42	
Distorted cognitions							*F* (1, 63) = 0.32
yes	4	48	0.13	0.71			
no	2	17	0.27	2.98^+^	0.14	0.56	
Cognitive beliefs							*F* (1, 63) = 1.99
yes	4	40	0.04	0.24			
no	2	25	0.31	2.75**	0.27	1.41	
Deviant peer association							*F* (1, 63) = 1.68
yes	3	29	0.35	2.53*			
no	3	36	0.10	0.73	-0.25	-1.30	
Peer problems							*F* (1, 63) = 2.63
yes	2	28	0.32	2.98**			
no	4	37	0.03	0.18	−0.29	1.62	
Parenting/family functioning							*F* (1, 63) = 10.54**
yes	3	37	0.03	0.42			
no	3	28	0.40	4.83***	0.37	3.25**	
Skills							*F* (1, 63) = 0.53
yes	5	56	0.05	0.18			
no	1	9	0.26	2.19*	0.21	0.73	
Social support (system)							*F* (1, 63) = 2.02
yes	2	21	0.31	2.75**			
no	4	44	0.03	0.22	−0.28	−1.42	
Structured free time							*F* (1, 63) = 1.68
yes	3	29	0.35	2.53*			
no	3	36	0.10	0.73	−0.25	−1.30	
Duration							*F* (1, 63) = 10.54**
6 months or less	3	37	0.03	0.42			
More than 6 months	3	28	0.40	4.83***	0.37	3.25**	
Level of program integrity							*F* (1, 63) = 4.81*
No mention of program integrity	3	31	0.11	1.28			
Program integrity medium to high	4	34	0.29	3.58***	0.18	2.19*	
Study design characteristics							
Study design							*F* (2, 62) = 1.40
RCT	2	21	0.04	0.22			
Matched	2	28	0.21	1.28	0.17	0.75	
Non-matched	2	16	0.43	2.54*	0.39	1.67	
Control condition							*F* (1, 63) = 0.00
TAU	5	57	0.22	1.78^+^			
No treatment	1	8	0.22	0.80	−0.00	−0.01	
Quality index	6	65	0.21	2.38*	−0.05	−1.83	*F* (1, 63) = 3.35^+^
Time of assessment							*F* (1, 63) = 0.69
Post-test	1	2	0.34	1.92^+^			
Follow-up test	6	63	0.22	2.12*	−0.12	−0.83	
Follow-up length	6	65	0.22	2.17*	0.00	0.28	*F* (1, 63) = 0.08
Publication characteristics							
Publishing year	6	65	0.21	2.45*	0.06	1.87^+^	*F* (1, 63) = 3.49^+^
Impact factor	4	43	0.12	1.79^+^	−0.12	−2.87**	*F* (1, 41) = 8.26**
Published							*F* (1, 63) = 0.86
yes	5	51	0.20	1.86^+^			
no	2	14	0.29	2.27*	0.09	0.93	

*Note. k* = number of studies; *#*ES = number of effect sizes; B_0_/mean *g* = intercept/mean effect size (Hedges’ *g*); t_0_ = *t*-value for mean *g*; B_1_ = regression coefficient or difference with intercept/reference category; t_1_ = *t*-value for regression coefficient; *F*(df1, df2) = omnibus test; TAU = treatment as usual.

1 All interventions targeted social problems, self-regulation, and coping, and none targeted knowledge through psycho-education nor moral outcomes (including moral judgment, empathy, and self-conscious emotions such as guilt and shame; see Heynen et al., 2023).

+ *p* < .10 (trend). *p < .05. **p < .01. ***p < .001.

### Moderator Analyses

Regarding *outcome characteristics*, a moderating effect of offense type (broad) was found, with intervention effects on general offenses (*g* = 0.28) being twice as large as effects on specific (property and violent) offenses (*g* = 0.14). As for *program characteristics*, we found significantly different effects across interventions. The mean effect for the social skills interventions TOOLS4U (*g* = 0.03), and the family-based systemic (parenting) interventions (*g* = 0.03; Multidimensional Family Therapy, Multi Systemic Therapy) was non-significant. The mean effect for the individual sports interventions (Only You Decide who you Are) was significant but small (*g* = 0.22), translating into a reduction of 12.36% in juvenile delinquency. The overall effect size for responsive CBT (Responsive Social Skills Therapy, Responsive Aggression Regulation Therapy for Young Adults) was medium to large (*g* = 0.50), translating into a reduction of 27.64% in criminal offense recidivism. The latter two interventions were merged into one category, because both are individually oriented CBT treatments following the RNR principles (in particular general and specific responsiveness) according to the study by Hoogsteder et al. (2015).

The analyses revealed that interventions addressing parenting/family functioning did not yield a significant mean effect (*g* = 0.03), whereas interventions without this target did yield a significant and small-to-medium effect (*g* = 0.40). We also found a moderating effect of treatment duration in the sense that treatment that lasted more than 6 months (*g* = 0.40) produced a significantly larger mean effect than treatment that lasted 6 months or less (*g* = 0.03, n.s.). However, treatment duration proved to be confounded with the moderating effect of the parenting/family functioning target of interventions, which may imply that only family-based systemic interventions of substantial duration may be effective. Regarding the program integrity of interventions, we found a difference in mean effect. Studies that reported a medium to high level of program integrity yielded a significant and small effect (*g* = 0.29), translating into a reduction of 16.24% in juvenile delinquency, which is significantly higher than the mean effect of studies that did not report on program integrity (*g* = 0.11). Of all *study design characteristics*, only the study quality index could be designated as moderator. As the quality of studies increases, the reported effects decrease (i.e., higher effects are reported in studies of lower quality).

No moderating effects were found for offense type (narrow), delinquency type, outcome type, mean age of samples, percentage of boys in samples, most of the coded program targets except parenting/family functioning, type of study design, nature of control condition, time of assessment, follow-up length, and whether or not studies have been published.

### Bias Assessment

**Figure A3.**
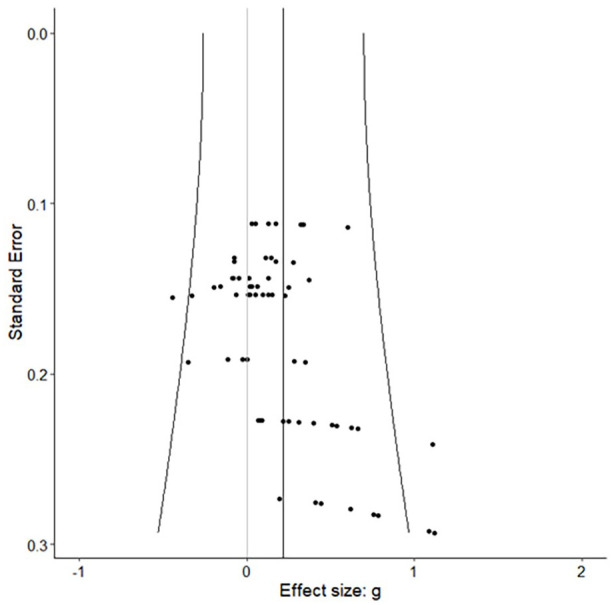
Trim-and-fill funnel plot.

**Figure A4.**
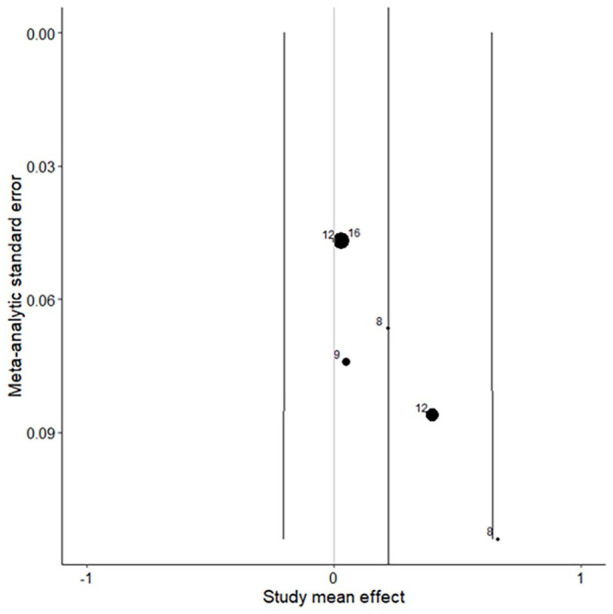
Funnel Plot of mean study effects, showing the number of effect sizes per study sample.

Publication and/or selection bias could not be ruled out, as our bias assessment strategy yielded inconsistent results. The adjusted Egger’s test showed that publication bias may have affected the results (*b* = 3.095, *t* = 2.560, *p* = .01). Visual inspection of the trim and fill plots suggested asymmetry at the right side of the study level distribution of effect sizes (see Figures A3 and A4), suggesting selection bias. This was confirmed in a formal test, indicating that two effect sizes were missing at the right side of the funnel plots. However, two effect sizes did not exceed the cutoff value for indications of selection bias as given by Fernández-Castilla et al. (2021). Finally, both the journal impact factor and study publication year were identified as moderators. Effect sizes increase as journal impact factors decrease, and larger effects are reported in more recently published studies. These results indicate a risk of bias and a decline effect, respectively.

## Discussion

This meta-analysis evaluated the overall effectiveness of all judicial behavioral interventions certified by the Netherlands Youth Institute (NYI) with at least *initial indications of effectiveness*. This was done by synthesizing primary studies on intervention effectiveness that assessed delinquency and/or recidivism as outcomes and had a control group in their design. The results indicate an overall significant small and positive effect on juvenile delinquency and recidivism for the six curative or general interventions that were summarized. Moderator analyses revealed that only the sports-based intervention OYD and responsive cognitive behavioral therapy (Re-SST and Re-ART) proved to be effective. Central to Re-SST and Re-ART is the *Risk, Need, and Responsivity (RNR) model* (Andrews et al., 2011; Bonta & Andrews, 2023). In addition to cognitive behavioral therapy, these interventions incorporate experiential exercises, mindfulness-based interventions (Hoogsteder et al., 2023), and involvement of the family system. Further, and in line with the RNR principles, a multimodal treatment approach is applied in these two interventions, that allow for the integration of additional treatments when necessary. Examples include the provision of psychiatric consultation and trauma therapy (Hoogsteder et al., 2022).

Social skills training (TOOLS4U) and family-based systemic (parenting) interventions (MDFT and MST) showed no evidence of effectiveness, although targeting family functioning in interventions was confounded with the moderating effect of treatment duration, which may imply that the duration of the family-based systemic interventions that were studied in the included primary studies (i.e., MDFT and MST) may have been too short for successfully preventing delinquency in the medium-to-high risk youth that were sampled in those studies. Further, studies with a medium-to-high level of program integrity yielded stronger effects than studies that did not report at all on program integrity. Offense type was also identified as moderator, with general delinquency showing a larger effect than specific (property and violent) offenses, which is not in line with results from the meta-analyses of De Vries et al. (2015), Piquero et al. (2016), and Wilson and Lipsey (2000). However, interventions having an effect on general offenses may also be partially effective for specific forms of delinquency, which explains the larger effect that we found for general offenses. After all, most (juvenile) delinquents do not specialize in a particular type of offense, and therefore tend to recidivate on various (general) offenses (Slotboom et al., 2023).

As for the different intervention programs that we synthesized, it can be concluded that multimodal and multi-elemental responsive CBT, as well as individual sports interventions did effectively prevent juvenile delinquency. This aligns with previous research showing that systemic and individual CBT approaches, which target multiple criminogenic risk factors, are effective among youth (Newton et al., 2016; Özabacı, 2011; Perkins et al., 2023). Lipsey’s (2009) meta-analysis on primary factors that characterize effective interventions for juvenile offenders concluded that CBT yielded the largest effects, which is in line with the general responsivity principle of the RNR model (Bonta & Andrews, 2023). Further, De Vries et al. (2015) found in their meta-analysis on effective elements of interventions for preventing persistent juvenile delinquency the largest effects for programs that are behaviorally oriented, and grounded in Bandura’s cognitive social learning theory (Bandura & Walters, 1963). The effect of individual sports interventions was slightly weaker than was previously found by Jugl et al. (2023) in their meta-analysis. They synthesized various forms of (preventive and curative) sports interventions, that were offered across various settings (community, schools, prisons, and sports clubs), and to individuals in a broad age range (7-40), whereas only one preventive sports intervention was eligible for inclusion in the current meta-analysis: Only You Decide who you Are. This intervention is implemented only at Dutch sports clubs and targets youth in the ages from 12-18. Notably, effects of preventive interventions tend to be smaller than effects of curative interventions, because most participants in preventive interventions will show positive outcomes without further intervention (Tanner-Smith et al., 2018).

As for the Dutch judicial context, it can be concluded that the exclusive use of social skills interventions, family-based systemic (parenting) interventions or parenting/family functioning interventions seems not effective in preventing or reducing juvenile delinquency. As for social skills interventions, meta-analytic evidence for long-term effects is lacking (Beelmann & Lösel, 2021; Van der Stouwe et al., 2021). However, juvenile offenders may still benefit from social skills training when integrated into multimodal preventive interventions, as is the case with Re-SST (see De Vries et al., 2015; Hoogsteder et al., 2021; Van der Stouwe et al., 2018). The lack of positive effects on juvenile delinquency of parenting interventions is in accordance with previous research, showing that effectiveness of such interventions tends to be short-term and rapidly declining after primary school age (Beelmann et al., 2023; Deković et al., 2011; De Vries et al., 2015; Latimer, 2001; Spruit et al., 2017; Tehrani et al., 2023; Van der Put et al., 2012). The effects of systemic interventions as examined in previous meta-analyses proved to be only small in the case of MDFT (Van der Pol et al., 2017), and the small effect of MST as reported by Van der Stouwe et al. (2014) was not found outside the USA and disappeared when effectiveness studies were conducted by independent researchers not involved in MST development. The recent systematic review by Littell et al. (2021) found no robust evidence for the effectiveness of MST, and even negative effects outside the USA.

Although confounded with the moderating effect of addressing the family system in interventions, interventions that last more than 6 months seem effective, whereas interventions with a duration of 6 months or less do not seem effective. This may indicate that (delinquent) youths with a medium-to-high risk of delinquency or criminal recidivism need longer and thus more intensive treatment of substantial length. This aligns with the RNR model emphasizing that more intensive treatment is needed as the risk of recidivism increases (Andrews et al., 2011; Bonta & Andrews, 2023). Interventions seem also more effective when implemented with a medium-to-high level of program integrity (*program integrity principle*; Andrews et al., 2011). This aligns with previous research showing positive effects of interventions with medium-to-high program integrity (Duwe & Clark, 2015; Hoogsteder et al., 2016; Van der Laan et al., 2010), which also holds for interventions that address juvenile antisocial (including delinquent) behavior in general (Goense et al., 2016).

The moderating effects of study quality index and journal impact factor are in accordance with research illustrating that articles and journals of lower quality and published in journals with a lower impact factor have less stringent requirements regarding for instance design and methodology, with larger effects as a result (Weisburd et al., 2001). The currently found larger effects of more recently published studies align with research showing that such studies are often based on the latest state-of-the-art interventions, incorporating recent advancements, which presumably produce larger effects (Beelmann & Lösel, 2021; Van Dam et al., 2020; Van der Put et al., 2021).

### Limitations

The present findings should be interpreted in light of several limitations, which are largely due to the inclusion of only those six interventions for which the NYI stated that *initial indications of effectiveness* are available. However, it was the particular aim of our meta-analysis to summarize the available evidence on the effects of these interventions to evaluate the program effectiveness in the Dutch context. Notably, many Dutch intervention developers have not submitted their intervention to the NYI for certification due to (for instance) lack of awareness about certification, time, or adequate funding. It is sometimes assumed by intervention developers, practitioners, and policy advisors that the certification process may delay the implementation of an intervention that seems promising in decreasing juvenile delinquency, while lack of certification does not necessarily imply ineffectiveness. Some have argued that numerous uncertified interventions proved to be effective in high-quality effectiveness studies, but still do not meet criteria for NYI certification, or just have never been submitted for certification. However, comprehensive searches by Spanjaard (2019) and Hanrath et al. (2023) and our own efforts yielded only a few uncertified Dutch judicial interventions that were evaluated in high-quality effectiveness studies, and generally showed no effect (De Vries et al., 2014), or even negative effects (e.g., Vooren et al., 2023).

Second, we excluded studies that did not report on juvenile delinquency or recidivism, including studies that reported on three interventions with positive effects on a multiple risk index (see for example Bout et al., 2017; Van Horn & Wilpert, 2017). One could argue that effectiveness of judicial interventions should not exclusively be based on the prevention of delinquency or criminal recidivism, but should also pertain to discovering talent, starting an education, or getting a job. However, we wanted to draw conclusions about the direct effect of judicial interventions on juvenile delinquency or recidivism (i.e., primary outcomes of judicial interventions) rather than on indirect intervention effects on risk or protective factors for juvenile delinquency (i.e., secondary outcomes of judicial interventions) or non-criminogenic needs. Third, our bias assessment strategy revealed that bias may have affected the current results. Notably, publication/selection bias can never be entirely ruled out in meta-analytic research in general and on intervention effectiveness in specific, unless all clinical intervention trials are preregistered.

## Conclusion

The current meta-analysis supports the use of two types of judicial behavioral interventions certified by the Netherlands Youth Institute (NYI) to effectively prevent juvenile delinquency and recidivism: responsive cognitive behavioral therapy (Responsive Social Skills Therapy and Responsive Aggression Regulation Therapy) and the sports-based intervention Only You Decide who you Are, which should be carried out with a substantial degree of program integrity to obtain the desired effects. However, the NYI *also* certified types of interventions (at the level of *initial indications of effectiveness*) that showed no evidence of effectiveness: social skills training (TOOLS4U) and family-based systemic (parenting) interventions (Multidimensional Family Therapy and Multi Systemic Therapy), which is unfortunate as the current results suggest that these interventions do not prevent (further) delinquent behavior in juveniles.

This review stresses the need for a stronger evidence-based approach to prevent and reduce juvenile delinquency in the Netherlands. Hendriks and Stams (2024) argue that only scientifically proven effective interventions should be certified and implemented. This aligns with the broader international context in which is noted that “the impact of evidence-based programs in juvenile justice programming is not yet what is hoped for,” and that suggests a more generic approach using knowledge about effective interventions through for example the Standardized Program Evaluation Protocol (SPEP; Elliott et al., 2020; Lipsey, 2008). Wilson and Lipsey (2024) also pay attention to a more generic approach, and similarly highlight that only a small percentage of rehabilitative programs for justice-involved youth are evidence-based. They advocate for designing programs that target effective levers of change (e.g., school engagement and family functioning) while avoiding addressing ineffective factors (e.g., academic achievement) to increase the likelihood of implementing programs that are effective.

The few interventions that could be designated as effective in the current meta-analysis raises doubts about whether the Dutch certification system, which requires interventions to be certified on the basis of robust effectiveness research, is an adequate policy instrument for achieving more evidence-based forensic youth care in the Netherlands. While only a few interventions seem effective according to the current results and treatment options for practitioners seem thus limited, large-scale implementation of these interventions could still yield major benefits at the population level (Elliott et al., 2020). Nevertheless, when no suitable certified intervention is available for which some scientific evidence of effectiveness is available, rather than developing new interventions that would add to the already growing number of unproven programs, it is more fruitful to adapt promising theory-based interventions in accordance with evidence-based principles for effective interventions and test their effectiveness in (quasi-)experimental research.

In doing so, attention to international effectiveness research is warranted, in particular because interventions may produce comparably positive results in different countries or continents (*cross-cultural transportability of psychosocial interventions*; Leijten et al., 2016; Maciel et al., 2023), although (sometimes) cross-cultural adaptations may be necessary (Van Cappellen et al., 2023). Foreign interventions should only be adopted if they are grounded in theory, were built on evidence-based principles for effective interventions, and have been tested in (quasi-)experimental research.

Practitioners should acknowledge the importance of an evidence-based approach to preventing (further) delinquency in juveniles effectively, and make research-informed decisions. To achieve this, building stronger partnerships between science and practice is needed. The Dutch national quality framework of effective youth interventions for preventing juvenile delinquency (Hendriks & Stams, 2024), the “Database Effective Youth Interventions” of the NYI that describes certified and currently monitored interventions, and the principles of the RNR model play a key role in strengthening effective strategies for practice. Moreover, high-quality primary research is warranted to strengthen and refine the evidence base for the RNR model, as a substantial amount of studies clearly support RNR (e.g., Bonta & Andrews, 2023; Hoogsteder et al., 2015), whereas other studies advocate more stringent testing of the RNR model than what is currently available (Bijlsma et al., 2022; Duan et al., 2024; Fazel et al., 2024). Future research should also evaluate the NYI database, along with its procedures for certification (which seems incorrect for ART given the lack of empirical support, see methods section) and monitoring, and identify gaps in the availability of Dutch interventions to better address the needs of juvenile offenders and at-risk juveniles.

## Appendix

**Table A1.** By the NYI Certified Judicial Youth Interventions Theoretically Founded for Effectiveness.

Judicial intervention (16)	Goal = preventing delinquency	Age	Certification level = theoretically founded for
“Grip on aggression”/Grip op Agressie (GOA)	Aggression / general delinquency / recidivism	18–25	General delinquency
“Training Aggression Control” / Training Agressie Controle (TACT)	Antisocial and aggressive behavior	12–23	General delinquency
“Training Sports and Behavior I” / Training Sport en Gedrag I	Slightly aggressive behavior on the football field	12–18	Minor violent crimes
“Training Sports and Behavior II” / Training Sport en Gedrag II	Aggressive behavior on the football field	12–18	Violent crimes
“Know your limit” / Ken je grens	Sexual misconduct	12–18	Sexual crimes
Out of the circle	Sexual misconduct	12–24	Sexual crimes
“Beware of the limit” / Pas op de grens	Sexual misconduct	12–21	Sexual crimes
Respect online	Sexual misconduct	12–18	Sexual crimes
Basta	Early signs of delinquency	12–	General delinquency
“Better start” / Betere start	Children of incarcerated mothers	2–10	General delinquency
“Diamond” / Diamant	Radicalization	12–23	Violent crimes
So Cool (educational penalty)	Educational penalty social skills slight mental handicap	12–23	General delinquency
“Relational family therapy” / Relationele gezinstherapie (RGT)	Behavior problems / delinquency / recidivism	12–17	General delinquency
“Top care” / Topzorg	Repeating offenders	17–23	Persistent general delinquency
“I don’t accept this” / Dit pik ik niet	Property crimes	12–18	Property crimes
TOPs! (sociomoral)	Antisocial behavior / delinquency / recidivism	12–24	General delinquency

**Table A2.** By the NYI Certified Judicial Youth Interventions With Initial Indications of Effectiveness.

Judicial intervention (9)	Type	Goal = preventing delinquency	Age	Certification level = initial indications for
TOOLS4U (educational penalty)	Curative, and preventive for first offenders and in the context of relapse (recidivism)	General delinquency / recidivism	12–23	General delinquency
“Multidimensional Family Therapy” / Multidimensionele familietherapie (MDFT) (system intervention)	Curative, and preventive for first offenders and in the context of relapse (recidivism)	General delinquency and substance use related / recidivism	12–24	General delinquency
“Multi Systemic Therapy” / Multisysteem Therapie (MST) (system intervention)	Curative, and preventive for first offenders and in the context of relapse (recidivism)	General delinquency / recidivism	10–18	Property crimes
“Forensic Outpatient System Therapy” / Forensische Ambulante Systeem Therapie (FAST) (system intervention)	Curative, and preventive for first offenders and in the context of relapse (recidivism)	General delinquency / recidivism	12–18	General delinquency and violent crimes
“Only You Decide who you Are” / Alleen Jij Bepaalt wie je Bent (AJB) (behavioral intervention with sports)	(selective) preventive	General delinquency	12–18	General delinquency
“Responsive Social Skills Therapy” / Sociale vaardigheidstraining op Maat (SOVA op Maat) (social skills treatment)	Curative, and preventive for first offenders and in the context of relapse (recidivism)	General delinquency / recidivism	15–21	General delinquency
“Parenting with Love and Boundaries” / Ouderschap met Liefde en Grenzen (OLG) (family intervention)	Curative, and preventive for first offenders and in the context of relapse (recidivism)	General delinquency / recidivism	12–18	Violent crimes
“Responsive Aggression Regulation Therapy for Youth” / Agressieregulatie op Maat Jeugd	Curative, and preventive for first offenders and in the context of relapse (recidivism)	Aggression / general delinquency / recidivism	12–16	Violent crimes
“Responsive Aggression Regulation Therapy for Young Adults” / Agressieregulatie op Maat Jongvolwassenen	Curative, and preventive for first offenders and in the context of relapse (recidivism)	Aggression / general delinquency / recidivism	16–24	Property crimes, violent crimes, and general delinquency

**Table A3.** By the NYI Certified Judicial Youth Interventions With Good Indications of Effectiveness.

Judicial intervention (1)	Goal = preventing delinquency	Age	Certification level = good indications for
Aggression replacement training (ART)	Aggression / violent crimes / recidivism	12–23	Violent crimes

**Table A4.** By the NYI Certified Non-judicial Youth Interventions.

Non-judicial intervention (36)	Goal = preventing behavioral problems	Age	Certification level = varying
Parenting training			
Incredible years (group parent training)	Oppositional or antisocial behavior disorder	3–8	Strong indications
Parent-Child Interaction Therapy (PCIT) (parent training)	Behavioral problems	2–7	Good indications
“Less angry and oppositional” / Minder boos en Opstandig (child- and parent training)	Behavioral problems	8–12	Good indications
Behavioral Parent Training Groningen Groep (BPTG-G)	ADHD and behavioral problems	8–12	Good indications
“Intensive Ambulant Family Treatment” / Intensieve Ambulante Gezinsbehande-ling (IAG)	Children with multiple and complex problems	0–23	Initial indications
Parent Management Training Oregon (PMTO)	Externalizing behavioral problems and hyperactivity	4–12	Initial indications
Basic Trust (attachment intervention)	Behavioral-, emotional-, and attachment problems	2–17	Initial indications
“Integrative Therapy for Attachment” / Integratieve therapie voor gehechtheid (ITGG)	Behavioral-, and attachment problems	0–27	Well-founded
“Family central” / Gezin central (GC)	Serious parenting and growing up problems	0–17	Well-founded
VIPP-SD positive parenting and sensitive discipline	Behavioral problems	0–6	Strong indications
Tripple P level 3	Behavioral- and parenting problems	2–16	Initial indications
Gordon training	Behavioral- and parenting problems	2–12	Well-founded
“Alcohol counseling for young people” / Adviesgesprek Alcohol jongeren	Alcohol related behavioral problems	12–23	Well-founded
“Talking with children” / Praten met kinderen	Mild externalizing problem behavior	10–15	Good indications
NIKA (attachment intervention)	Serious behavioral and attachment problems	0–6	Well-founded
School programs			
“Alternative thinking strategies program” / Programma Alternatieve Denkstrategieën (PAD)	Behavioral problems	4–12	Strong indications
School2care	Complex and serious behavioral problems early school leavers	12–17	Well-founded
“Everything okay” / Alles Kidzzz	Externalizing behavior	9–12	Good indications
School wide Positive behavioral support	Behavioral problems and social behavior on school	4–18	Well-founded
Kwink	Classroom climate	4–12	Well-founded
“Power and control” / Kracht en Controle	Social competence pre-vocational secondary education students	11–14	Well-founded
Be wise, think twice	Problematic substance use	12–14	Well-founded
“Bright at school: fresh together” / Helder op school: samen fris	Problematic substance use	13–15	Well-founded
Join us (class climate)	Bullying at school	11–13	Well-founded
“Superheroes” / Superhelden	Group climate class	4–12	Well-founded
Futsall Chabbab	Reducing school dropout	8–18	Well-founded
Kiva	Bullying at school	4–12	Strong indications
“Fine” / Prima	Bullying at school	4–13	Initial indications
Other programs			
Stop 4–7	Serious behavioral problems / antisocial behavior	4–7	Initial indications
Take it personal	Problematic substance use slight mental handicap	10–27	Well-founded
“Standing Strong Together” / Samen Stevig Staan	Slight mental handicap with behavioral problems / disorders	9–16	Well-founded
Youth Empowerment Through Sports (YETS) (intervention with sports)	Preventing behavioral problems and social dropout	12–18	Well-founded
“PBS in the neighborhood” / PBS in de wijk	Behavioral and social problems	2–18	Well-founded
“Open and alert” / Open en alert	Problematic substance use	12–25	Well-founded
Moti-4	Substance use, gambling, and gaming	12–14	Good indications
Unity (information program)	Alcohol and drugs	19–35	Well-founded

**Table A5.** Characteristics of Included Studies.

Author (s)	Pub year	Program name	*N*	Mean age	Design	Matched	Control group	Offense type	Delinquency type	Delinquency assessment	Outcome type	*#ES*
Asscher et al.	2013 2014	Multi Systemic Therapy	256	16.02	RCT	No	TAU	General, violence, property	Arrest, self-reported delinquency	Official record, questionnaire	Percentage, time to first, number	12
Gubbels et al.	2018	Only You Decide who you Are	364	13.93	QE	No	NT	General	Arrest, convicted	Official record	Percentage, number	8
Hoogsteder et al.	2018	Responsive Aggression Regulation Therapy for Young Adults	91	16.89	QE	Yes	TAU	General, violence, property	Convicted	Official record	Number	12
Hoogsteder et al.	2021	Responsive Social Skills Therapy	57	17.07	QE	No	TAU	General, violence, property	Convicted	Official record	Percentage	8
Van der Pol et al.	2018 2021	Multidimensional Family Therapy	109	16.80	RCT	Yes	TAU	General, violence, property	Arrest	Official record	Time to first, severity, number	9
Van der Stouwe et al.	2019 2020	TOOLS4U	172	15.73	QE	Yes	TAU	General, violence	Convicted	Official record	Percentage, time to first, number	10
Van der Stouwe et al.	2018	TOOLS4U	230	16	QE	Yes	TAU	General	Court/police contact	Official record	Percentage	6

*Note*. Design = randomized controlled trials (RCT), quasi-experimental (QE); Control group = no treatment (NT), treatment as usual (TAU); *N* = sample size total (start of the study); Delinquency type = arrest, convicted, court/police contact, self-reported delinquency; Offense type = general, violence, property; Delinquency assessment = official record, questionnaire self-report; Outcome type = percentage offenses, time to first offense, severity of offense, number of offenses; *#ES* = number of effect sizes.
